# Exocarpium Citri Grandis Attenuates Lipopolysaccharide‐Induced Acute Liver Injury Through Suppression of Inflammatory, Apoptotic, Oxidative, and Ferroptotic Pathways

**DOI:** 10.1002/fsn3.71012

**Published:** 2025-09-26

**Authors:** Guoni Zhang, Jiayu Li, Zaibin Xu, Kaili Zhou, Xinyi Zhao, Xiaoqin Wu, Chao Liang, Zhijun Lai, Yi Cheng, Cong Meng, Jiawen Huang, Zhitong Deng

**Affiliations:** ^1^ Haikou Hospital of Traditional Chinese Medicine Haikou China; ^2^ Science and Technology Innovation Center Guangzhou University of Chinese Medicine Guangzhou Guangdong China; ^3^ The Fifth Clinical Medical College of Guangzhou University of Chinese Medicine Guangzhou China; ^4^ The Affiliated TCM Hospital of Guangzhou Medical University Guangzhou China

**Keywords:** anti‐inflammatory, apoptosis and ferroptosis, *Exocarpium Citri Grandis*, LPS‐induced acute liver injury, network pharmacology

## Abstract

This study aimed to elucidate the therapeutic mechanisms of Exocarpium Citri Grandis (ECG) against lipopolysaccharide (LPS)‐induced acute liver injury (ALI) using an integrated approach combining network pharmacology and experimental validation. The chemical profile of ECG was characterized via UHPLC‐Q‐Exactive MS. Male C57BL/6J mice were employed to establish an LPS‐induced ALI murine model for evaluating hepatoprotective effects of ECG. Serum AST/ALT levels, histopathology changes, and inflammatory, apoptotic, oxidative, and ferroptotic markers were analyzed. Additionally, network pharmacology was used to identify the targets and pathways of ECG, while molecular docking validated interactions between bioactive compounds and key regulatory molecules. ECG treatment significantly reduced serum AST/ALT levels (*p* < 0.05–0.001) and alleviated hepatic inflammation and necrosis. Moreover, qPCR and Western blotting revealed that ECG markedly downregulated pro‐inflammatory cytokines (IL‐6, IL‐1β, TNFα), inhibited cellular inflammation by downregulating the expression of proteins related to the TLR4/MyD88 pathway, counteracted oxidative stress by increasing Nrf2 nuclear translocation and the expression of genes such as NQO1, GCLC, and GCLM, suppressed apoptosis via modulation of Bax/Bcl2/CASP3, and inhibited ferroptosis by normalizing iron content and GPX4/SLC7A11 expression. Network pharmacology identified 127 shared targets between ECG and sepsis, highlighting critical pathways including TNF, p53, and IL‐17 signaling. Molecular docking confirmed strong binding affinities (e.g., naringin with TP53: −13.23 kcal/mol) between ECG‐derived flavonoids and coumarins with apoptosis and inflammation regulators. ECG mitigates LPS‐induced ALI through coordinated anti‐inflammatory, anti‐apoptotic, anti‐oxidative, and anti‐ferroptotic mechanisms, mediated via multi‐target modulation of critical pathways. These findings underscore the potential of ECG as a natural therapeutic candidate for ALI, thus warranting further clinical exploration.

## Graphical abstract

ECG mitigates LPS‐induced ALI through coordinated anti‐inflammatory, anti‐apoptotic, and anti‐ferroptotic mechanisms, supported by multi‐target modulation of critical pathways.

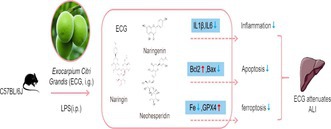

**Abbreviations**
ALI: Acute liver injury
APAP: acetaminophen
ECG: Exocarpium Citri Grandis
LPS: lipopolysaccharide
NAFLD: nonalcoholic fatty liver disease
RNS: reactive nitrogen species
ROS: reactive oxygen species
SD: standard deviation
TCM: traditional Chinese medicine

## 1 Introduction

Acute liver injury (ALI) is a life‐threatening condition characterized by rapid hepatocyte necrosis, inflammation, and hepatic dysfunction, often progressing to liver failure if left untreated. Common etiologies include drug toxicity (e.g., acetaminophen overdose), viral infections, metabolic disorders, and sepsis‐induced endotoxemia (Peng et al. 2019; Ozturk et al. 2023). Among these, bacterial lipopolysaccharide (LPS), a key component of gram‐negative bacterial cell walls, is a potent inducer of systemic inflammation and organ damage, including ALI (Zhang et al. 2020). LPS activates Toll‐like receptor 4 (TLR4) on Kupffer cells, triggering the release of pro‐inflammatory cytokines (e.g., TNFα, IL‐6, IL‐1β), reactive oxygen species (ROS), and nitrogen species (RNS), which collectively drive hepatocyte apoptosis, ferroptosis, and necroinflammation (Zhao et al. 2023; Radi 2004). Despite advances in supportive care, current treatments for ALI remain limited, with liver transplantation as the sole curative option for end‐stage cases (Monti et al. 2016). This underscores an urgent need for novel therapies that target multiple pathogenic pathways while minimizing adverse effects.

Natural products have re‐emerged as promising candidates for treating complex diseases, owing to their multi‐component, multi‐target therapeutic profiles. Unlike synthetic drugs, which often act on single pathways, natural compounds synergistically modulate interconnected biological networks, thereby offering broader efficacy and a lower risk of resistance (Atanasov et al. 2021). Exocarpium Citri Grandis (ECG), derived from the peel of *Citrus grandis* and historically used in traditional Chinese medicine for respiratory disorders, exemplifies this potential. Rich in flavonoids (e.g., naringin, neohesperidin) and coumarins, ECG has been reported to exhibit anti‐inflammatory, antioxidant, and anti‐ferroptotic activities (Jiang et al. 2014; Xu et al. 2024). Recent studies demonstrate its efficacy in alleviating LPS‐induced lung injury and nonalcoholic fatty liver disease (NAFLD) by regulating lipid metabolism and iron homeostasis (Zhu et al. 2019; Deng et al. 2023). However, its mechanisms in ALI, particularly in modulating apoptosis and ferroptosis, remain unexplored.

To address this gap, we employed an integrated approach combining network pharmacology and experimental validation. Network pharmacology is a systems biology‐driven method uniquely suited for studying traditional Chinese medicine (TCM) because it maps compound‐target‐pathway interactions and bridges the gap between holistic TCM principles and reductionist molecular biology (Li et al. 2023). Here, we hypothesized that ECG protects against LPS‐induced ALI through dual suppression of inflammatory and cell death pathways. To test this hypothesis, we utilized a murine LPS‐induced ALI model. This model is well established for studying sepsis‐associated liver injury due to its reproducibility and relevance to human endotoxemia pathophysiology (Chen et al. 2021). Male C57BL/6J mice were selected for their genetic homogeneity and robust immune responses, which ensure consistency in evaluating ECG's dose‐dependent effects (Xu et al. 2024). Although this model primarily mimics sepsis‐driven ALI, conserved pathways such as TLR4/NF‐κB signaling and p53‐mediated apoptosis are shared across species. This enhances translational relevance to human conditions like drug‐induced or viral hepatitis‐associated ALI (Zhao et al. 2024; Li et al. 2021).

In this study, we characterized the chemical profile of ECGs, validated its hepatoprotective effects in vivo, and deciphered its mechanisms through network pharmacology and molecular docking. Our findings not only advance the understanding of ECGs' multi‐target actions but also provide a framework for developing natural product‐based therapies for ALI.

## 2 Materials and Methods

### 2.1 UHPLC‐Q‐Exactive Analysis

The chemical constituents of ECG were characterized using ultra‐high‐performance liquid chromatography coupled with quadrupole‐Exactive mass spectrometry (UHPLC‐Q‐Exactive MS) as described in our previous study (Xu et al. 2024). Briefly, 100 mg of lyophilized ECG powder was homogenized in methanol (1 mL) using a cryogenic grinder at −40°C for 2 min. After ultrasonic extraction in an ice‐water bath for 1 h, the mixture was centrifuged at 12,000 rpm for 10 min. The supernatant was filtered through a 0.22 μm membrane and diluted 10‐fold prior to analysis. Chromatographic separation was performed on an ACQUITY UPLC HSS T3 column (100 × 2.1 mm, 1.8 μm) with a mobile phase comprising 0.1% formic acid (A) and acetonitrile (B). The gradient elution program was optimized as follows: 0–2 min, 95% A; 2–15 min, 95%–0% A; 15–16 min, 95% A. Mass spectrometry detection was conducted in both positive and negative ion modes using a HESI source, with full MS/dd‐MS^2^ (TOP 8) scanning. Compound identification was validated by comparing experimental m/z values with theoretical masses from the TCM Integrated Database (TCMID, http://www.megabionet.org/tcmid/) and PubChem, with a mass error threshold of < 5 ppm.

### 2.2 Animal

Animal experiments were conducted in accordance with the ARRIVE guidelines (McGrath and Lilley 2015; Percie du Sert et al. 2020) and approved by the Ethics Committee of Guangzhou University of Chinese Medicine (No. 20240923013). Male C57BL/6J mice (8 weeks old, 22–25 g) were obtained from Guangdong Medical Laboratory Animal Center and acclimatized for 1 week under specific pathogen‐free (SPF) conditions (26°C ± 1°C, 12 h light/dark cycle) with ad libitum access to standard chow and filtered water in the Animal Experiment Center, Guangzhou University of Chinese Medicine (No. 00436493). To minimize environmental bias, cages were arranged in a checkerboard pattern and rotated clockwise weekly.

The sample size for this study was determined a priori using a power analysis based on the primary outcome measure, serum alanine aminotransferase (ALT) levels, which is a key biomarker of LPS‐induced liver injury. The following parameters were used: Effect Size: Cohen's *d* = 2.54 (estimated from our previous study (Xu et al. 2024)). Significance Level: *α* = 0.05 (two‐tailed). Desired Power: 1 − *β* = 0.80. Allocation Ratio: 1:1 (Model vs. ECG‐H comparison). Using G*Power 3.1 (ANOVA: Fixed effects, omnibus, one‐way), the calculated sample size for detecting this large effect size (*f* = 1.27) with 80% power was *n* = 4 per group. To account for potential attrition and variability in biological systems, we increased the sample size to *n* = 5 per group for all four experimental groups (Control, Model, ECG‐L, ECG‐H).

Mice were randomly assigned to four groups (*n* = 5 per group) using a random number table: Control (Ctr): Oral administration of saline for 7 days. Model (Mod): Oral saline for 7 days + intraperitoneal (i.p.) LPS (5 mg/kg, *Escherichia coli* O55:B5, Sigma‐Aldrich) on day 7. ECG‐L (Low dose): Oral ECG (50 mg/kg/day) for 7 days + i.p. LPS. ECG‐H (High dose): Oral ECG (100 mg/kg/day) for 7 days + i.p. LPS.

LPS was dissolved in sterile saline and administered 6 h before euthanasia to induce acute liver injury. Animals were monitored every 2 h post‐LPS injection for adverse reactions (e.g., lethargy, respiratory distress). Severe lung injury leading to acute respiratory distress syndrome (ARDS) was predefined as the humane endpoint; no animals reached this endpoint during the study.

Six hours post‐LPS challenge, mice were anesthetized with sodium pentobarbital (50 mg/kg, i.p.), and blood was collected via retro‐orbital puncture. Subsequently, euthanasia was performed by rapid cervical dislocation. Liver tissues were harvested, rinsed in ice‐cold PBS, and divided into aliquots for histopathology, molecular analyses, and iron content measurement. All procedures were performed by investigators blinded to group allocation, while the corresponding authors were aware of the group allocation at the different stages of the experiment.

### 2.3 Serum AST and ALT Measurement

The mice were then anesthetized by intraperitoneal injection of sodium pentobarbital (50 mg/kg). After 30 min, mouse blood was collected using the retro‐orbital puncture method. Subsequently, the mice were sacrificed via rapid cervical spine dislocation, and other tissues required for the assay were collected. Serum aspartate aminotransferase (AST) and alanine aminotransferase (ALT) levels were measured using a commercial assay kit. The measurements were performed following the instructions provided by the manufacturer (Nanjing Jiancheng Bioengineering Institute, China).

### 2.4 Hematoxylin Eosin (H&E) Staining

Tissues were fixed, embedded, and sectioned into 5 μm slices. Sections were then stained with H&E for histological examination. Images were captured using a light microscope (Nikon, Shanghai, China).

### 2.5 Immunohistochemistry

Paraffin‐embedded tissue sections (5 μm) were dewaxed with xylene, rehydrated, and subjected to antigen retrieval. Subsequently, blocking was performed with 5% goat serum at 37°C for 30 min. Primary antibodies (anti‐CD68, anti‐GPX4) were added to the sections and incubated at 4°C overnight. On the following day, after rinsing with PBS, secondary antibodies conjugated with horseradish peroxidase (HRP) were added to the sections and incubated at 37°C for 1 h. The sections were counterstained with hematoxylin to highlight cell nuclei. After dehydration with gradient ethanol and clearing with xylene, the sections were mounted with neutral mounting medium. Images were captured using a light microscope (Nikon, Shanghai, China) for subsequent histological analysis.

### 2.6 Immunofluorescence

Paraffin‐embedded tissue sections (5 μm) were dewaxed with xylene, rehydrated, and subjected to antigen retrieval. Subsequently, blocking was performed with 5% goat serum at 37°C for 30 min. Primary antibodies (anti‐p‐p65, anti‐CAS3, and anti‐Bcl‐2) were added to the sections and incubated at 4°C overnight. On the following day, after rinsing with PBS, the sections were incubated with fluorescein‐conjugated secondary antibodies (e.g., FITC‐conjugated, Cy3‐conjugated) at 37°C for 1 h. After another round of PBS rinsing, the sections were stained with 4′,6‐diamidino‐2‐phenylindole (DAPI) to highlight cell nuclei. Following dehydration with gradient ethanol and clearing with xylene, the sections were mounted with anti‐fluorescence quenching mounting medium. Images were captured using a fluorescence microscope (Nikon, Shanghai, China) for subsequent histological analysis.

### 2.7 Quantitative Real‐Time PCR (qPCR)

Total mRNA was extracted using Invitrogen TRIzol Reagent (Thermo Fisher Scientific Inc., USA). cDNA synthesis was then performed with the PrimeScript RT Reagent Kit (Takara Biomedical Technology (Beijing) Co. Ltd., China). Amplification of target genes was carried out using the CFX Connect Real‐Time PCR Detection System (Bio‐Rad Laboratories Inc., USA). The primers used for amplifying the target genes were listed in Table 1.

**TABLE 1:** Primers used in qPCR analysis.

Gene	Forward primer	Reward primer
IL1β	CCGTGGACCTTCCAGGATGA	GGGAACGTCACACACCAGCA
IL6	TAGTCCTTCCTACCCCAATTTCC	TTGGTCCTTAGCCACTCCTTC
Tnfa	CCCTCACACTCAGATCATCTTCT	GCTACGACGTGGGCTACAG
Bcl2	GACAAGGAGATGCAGGTATTGG	TCCCGTAGAGATCCACAAAAGT
Bax	TGAAGACAGGGGCCTTTTTG	AATTCGCCGGAGACACTCG
Fth	CAAGTGCGCCAGAACTACCA	GCCACATCATCTCGGTCAAAA
Slc7a11	GGCACCGTCATCGGATCAG	CTCCACAGGCAGACCAGAAAA
SOD2	CAGACCTGCCTTACGACTATGG	CTCGGTGGCGTTGAGATTGTT
β‐Actin	GGCTGTATTCCCCTCCATCG	CCAGTTGGTAACAATGCCATGT

### 2.8 Liver Tissue Iron Content

The iron content in liver tissues was measured using a commercial reagent kit (Nanjing Jiancheng Bioengineering Institute, China). Concurrently, protein concentrations were determined using a kit from Beyotime Biotechnology (China). The iron content of each tissue sample was normalized to its corresponding protein concentration for standardization.

### 2.9 Western Blotting

RIPA lysis buffer was used for protein extraction. Subsequently, proteins from samples were separated by SDS‐PAGE gel and transferred to PVDF membranes. After blocking, membranes were incubated with primary antibody overnight at 4°C, followed by incubation with secondary antibody at room temperature for 1 h. Images were collected using an ECL assay kit via the BIO‐RAD ChemiDoc system. Antibodies were purchased from Affinity Biosciences (USA).

### 2.10 Establishment of the ECG‐Ingredient–Target Interaction

Active ingredients associated with epicatechin gallate (ECG) were identified using the Traditional Chinese Medicine Systems Pharmacology Database (TCMSP) with the keyword “Exocarpium *Citri grandis*”. Ingredients were selected using the criteria of oral bioavailability (OB) ≥ 30% and drug‐likeness (DL) ≥ 0.18. The target genes associated with these ingredients were retrieved from TCMSP and Swiss Target Prediction (http://www.swisstargetprediction.ch/). Targets related to sepsis were obtained from several databases including DisGeNET (https://www.disgenet.org/), DrugBank (https://go.drugbank.com/drugs), GeneCards (https://www.genecards.org/), Open Targets Platform (https://platform.opentargets.org/), Online Mendelian Inheritance in Man (OMIM, https://www.omim.org/), and the Therapeutic Target Database (TTD, https://db.idrblab.net/ttd/). The UniProt database (http://www.uniprot.org) was utilized to convert these targets to gene identifiers, eliminating duplicate entries. Overlapping targets of ECG and sepsis were identified using the Venny (Draw Venn Diagram) tool (http://bioinfogp.cnb.csic.es/tools/venny/index.html).

### 2.11 Construction of a Protein–Protein Interaction (PPI) Network

These overlapping targets were further analyzed using the STRING database (https://cn.string‐db.org/cgi/input.pl) to construct a PPI network. The network was visualized using Cytoscape software (version 3.8.0).

### 2.12 Gene Ontology Enrichment and Pathway Analysis

To elucidate the potential mechanisms of ECG, the overlapping targets were analyzed using the DAVID database (http://david.ncifcrf.gov/, ver. 6.8) for gene ontology (GO) functional annotation analysis and Kyoto Encyclopedia of Genes and Genomes (KEGG) pathway analysis. The significance threshold was set to *p* < 0.05. The analysis focused on the enrichment of biological processes (BP), cellular components (CC), molecular functions (MF), and KEGG pathways. Results were prioritized in descending order of significance and were visualized using the SRplot web server (http://www.bioinformatics.com.cn/SRplot).

### 2.13 Molecular Docking

Molecular docking is a widely used method to predict binding interaction patterns between active ingredients and disease targets. The two‐dimensional (2D) and three‐dimensional (3D) structures of active ingredients from ECG were retrieved from the PubChem database (https://pubchem.ncbi.nlm.nih.gov/). Crystal structures of sepsis‐related targets were obtained from the RCSB Protein Data Bank (https://www.rcsb.org/). Molecular interactions between the active ingredients of ECG and sepsis‐related targets were analyzed using SYBYL‐X 2.2.1 software.

### 2.14 Statistical Analysis

All data are presented as the mean ± standard error of the mean (SEM). Statistical analyses were performed using SPSS 17 (IBM Corp., USA). Group differences were evaluated by one‐way analysis of variance (ANOVA) followed by Bonferroni's post hoc test for multiple comparisons, with a significance threshold set at *p* < 0.05.

Post hoc Power Analysis: Given the limited sample size (*n* = 5 per group), a post hoc power analysis was conducted using G*Power 3.1 (Heinrich Heine Universität Düsseldorf, Germany) to assess the risk of Type II errors. This analysis focused on 11 key parameters including ALT, AST, IL‐6, IL‐1β, TNFα, BAX, BCL2, Fe, SLC7A11, FTH, and SOD2. Statistical power was calculated under a Bonferroni‐adjusted significance level (*α* = 0.0045) to account for multiple comparisons. A power value ≥ 0.80 was predefined as indicative of sufficient statistical sensitivity, reflecting a low risk of Type II errors.

## 3 Results

### 3.1 Chemical Composition of ECG Extract

UHPLC‐Q‐Exactive MS analysis identified 15 major bioactive constituents in ECG, predominantly flavonoids and coumarins (Figure S1). Key flavonoids included naringin (m/z 579.1712 [M − H]^−^), neohesperidin (m/z 609.1825 [M − H]^−^), and naringenin chalcone (m/z 273.0758 [M + H]^+^), which are recognized for their anti‐inflammatory and antioxidant properties. Coumarin derivatives such as bergapten (m/z 217.0495 [M + H]^+^) and auraptene (m/z 299.1281 [M + H]^+^) were also detected. Additionally, the analysis revealed the presence of stachydrine (m/z 144.1019 [M + H]^+^), a pyrrolidine alkaloid with potential hepatoprotective effects. These findings corroborate ECG's multi‐component nature, providing a chemical basis for its observed dual anti‐inflammatory and anti‐ferroptotic activities in LPS‐induced liver injury.

### 3.2 ECG Mitigates LPS‐Induced ALI

**FIGURE 1:**
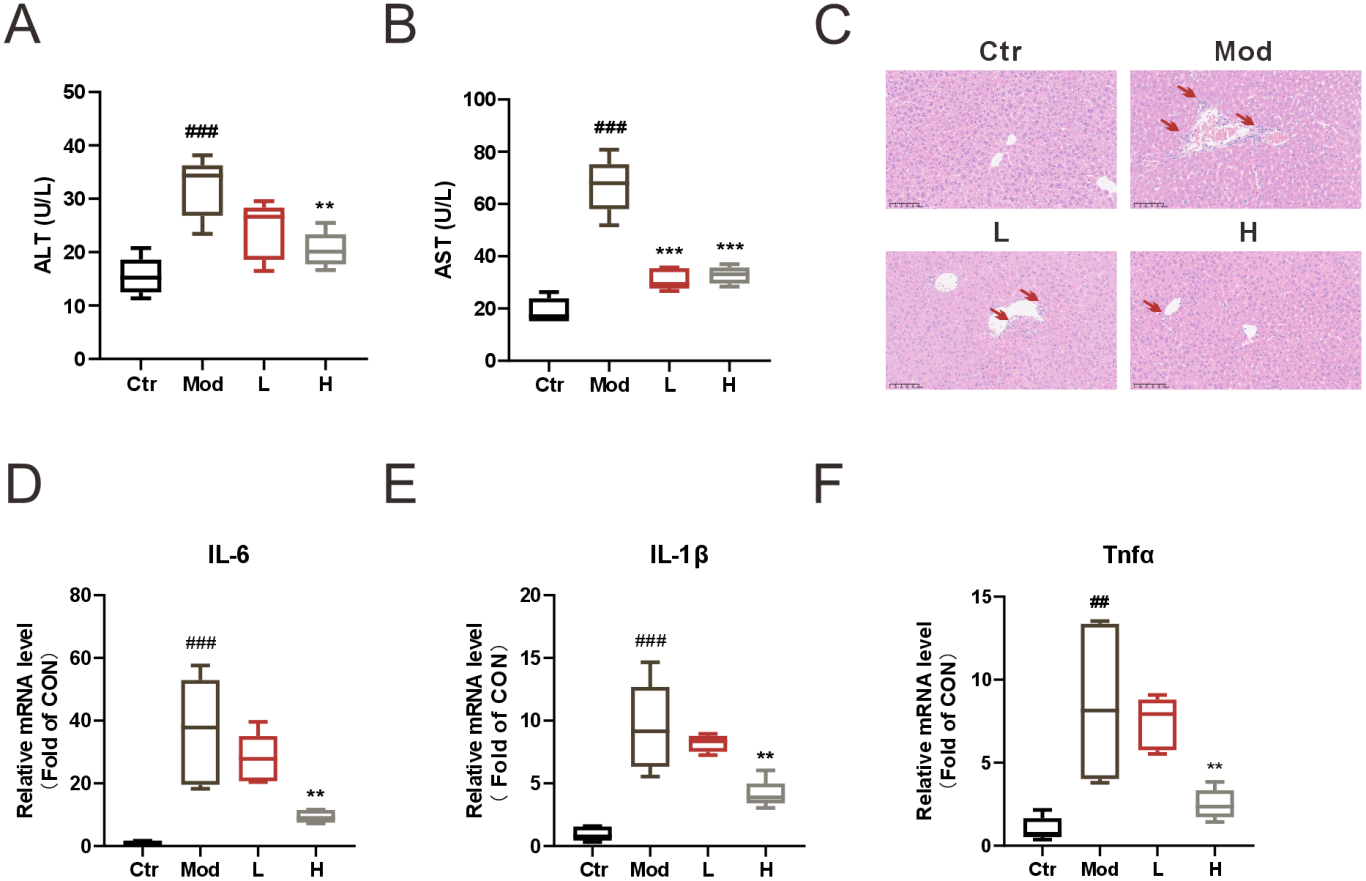
ECG prevents liver from acute liver injury in LPS‐induced mice. Serum ALT (A) and AST (B) levels; Representative pathological images of liver (C); Relative mRNA levels of IL‐6 (D), IL‐1β (E), and TNFα (F) in the liver. Data were presented as mean ± SEM (*n* = 5). ^##^ *p* < 0.01, ^###^ *p* < 0.001 vs. the Ctr group. **p < 0.01, and ***p < 0.001 vs. the Mod group.

All animals completed the experiment and were included in the statistics with no exclusions. As shown in Table S1, most parameters exhibited high statistical power. ECG's protective effect against LPS‐induced acute liver injury was assessed by measuring serum AST and ALT, which are key indicators of liver injury. LPS administration elevated serum AST (Figure 1A) and ALT (Figure 1B) levels, whereas ECG treatment significantly reduced these levels. Histopathological analysis revealed that compared to the control group, the model group exhibited severe damage to hepatic lobules, including edema and infiltration of inflammatory cells. ECG treatment ameliorated these pathological changes (Figure 1C). Additionally, mRNA levels of inflammatory markers such as IL‐6, IL‐1β, and TNFα were quantified via qPCR. LPS significantly increased these mRNA levels, while ECG treatment tended to reduce them (Figure 1D–F), suggesting that ECG protects against LPS‐induced ALI in mice.

### 3.3 ECG Inhibits Inflammation in LPS‐Induced Mice

**FIGURE 2:**
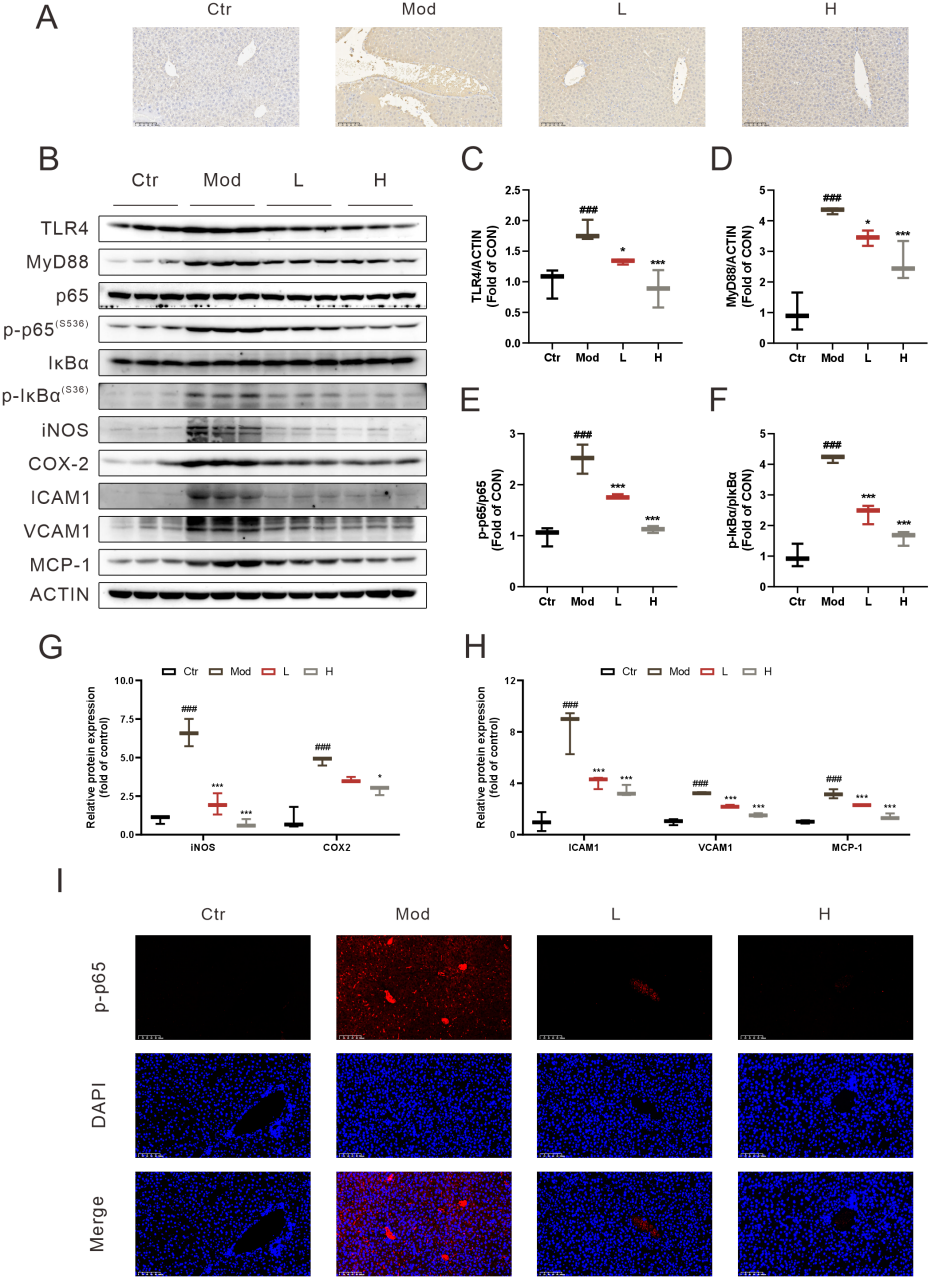
ECG inhibits inflammation in LPS‐induced mice. (A) Representative images of CD68 immunohistochemistry in the liver; (B) representative WB bands of TLR4, MyD88, p65, p‐p65, IκBα, p‐IκBα, iNOS, COX‐2, ICAM1, VCAM1, and MCP‐1 in liver; (C–H) quantitative analysis of WB bands; (I) represen tative images of p‐p65 immunofluorescence in the liver (I). Data were presented as mean ± SEM (*n* = 3–5).^###^ *p* < 0.001 vs. the Ctr group. *p < 0.05, ***p < 0.001 vs. the Mod group.

To evaluate ECG's effect on LPS‐induced hepatic inflammatory responses, inflammatory markers were further assessed. As shown in Figure 2A, LPS administration significantly increased CD68 expression, which was effectively reduced by ECG treatment. Western blot analysis (Figure 2B–H) revealed that compared with the control group, hepatic protein expression of TLR4, MyD88, p‐P65, p‐IκBα, COX‐2, ICAM1, VCAM1, and MCP1 was significantly upregulated in the model group. These increases were significantly inhibited by ECG intervention. Additionally, immunofluorescence assay (Figure 2I) showed that p‐P65 levels were significantly elevated following LPS treatment, with a decreasing trend observed after ECG administration. These findings indicate that ECG can effectively prevent LPS‐induced acute liver injury in mice.

### 3.4 ECG Inhibits Apoptosis in LPS‐Induced Mice

**FIGURE 3:**
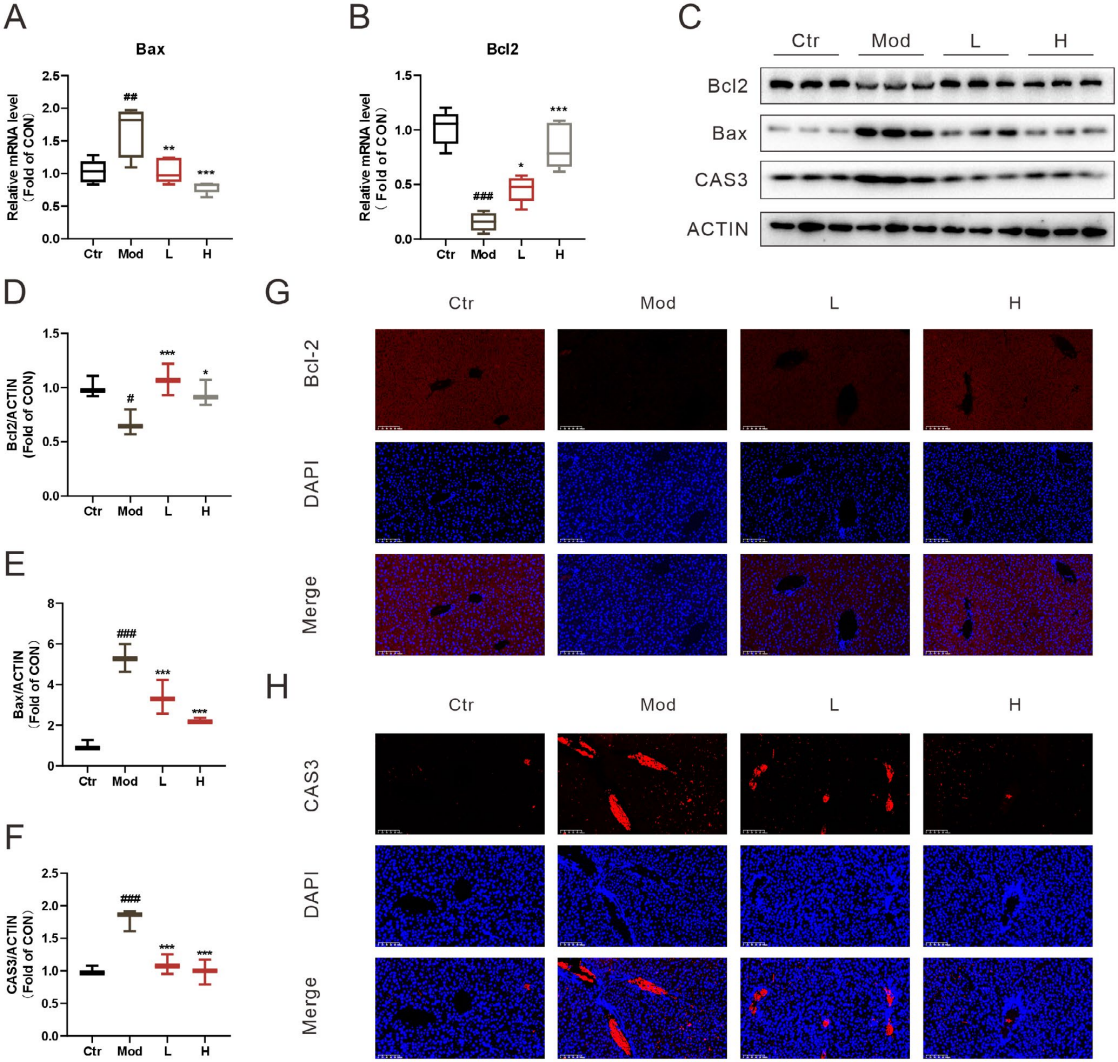
ECG inhibits apoptosis in LPS‐induced mice. Relative mRNA levels of Bax (A) and Bcl2 (B) in the liver; representative WB bands of Bcl2, BAX, CAS3 (C); quantitative analysis of WB bands (D–F); representative images of Bcl‐2, CAS3 immunofluorescence in the liver (G, H). Data were presented as mean ± SEM (*n* = 3–5). ^#^ *p* < 0.05, ^##^ *p* < 0.01, ^###^ *p* < 0.001 vs. the Ctr group. *p < 0.05, **p < 0.01, ***p < 0.001 vs. the Mod group.

Apoptosis is a hallmark feature of liver pathology in LPS‐induced ALI as documented in recent studies (Chen et al. 2021). In our investigation, elevated mRNA levels of pro‐apoptotic Bax and reduced mRNA levels of anti‐apoptotic Bcl2 were noted in the model group. Notably, treatment with ECG significantly reduced these mRNA levels (Figure 3A,B). Moreover, the LPS intervention reduced the expression of BCL2 protein and increased the expressions of BAX and CAS3 proteins while ECG treatment restored these abnormal levels (Figure 3C–F). Immunofluorescence analysis results showed that LPS led to the downregulation of Bcl‐2 and the upregulation of CAS3, whereas ECG markedly reversed these abnormal changes (Figure 3G–H). These results suggest that ECG effectively inhibits hepatic apoptosis in LPS‐induced mice.

### 3.5 ECG Inhibits Oxidation in LPS‐Induced Mice

**FIGURE 4:**
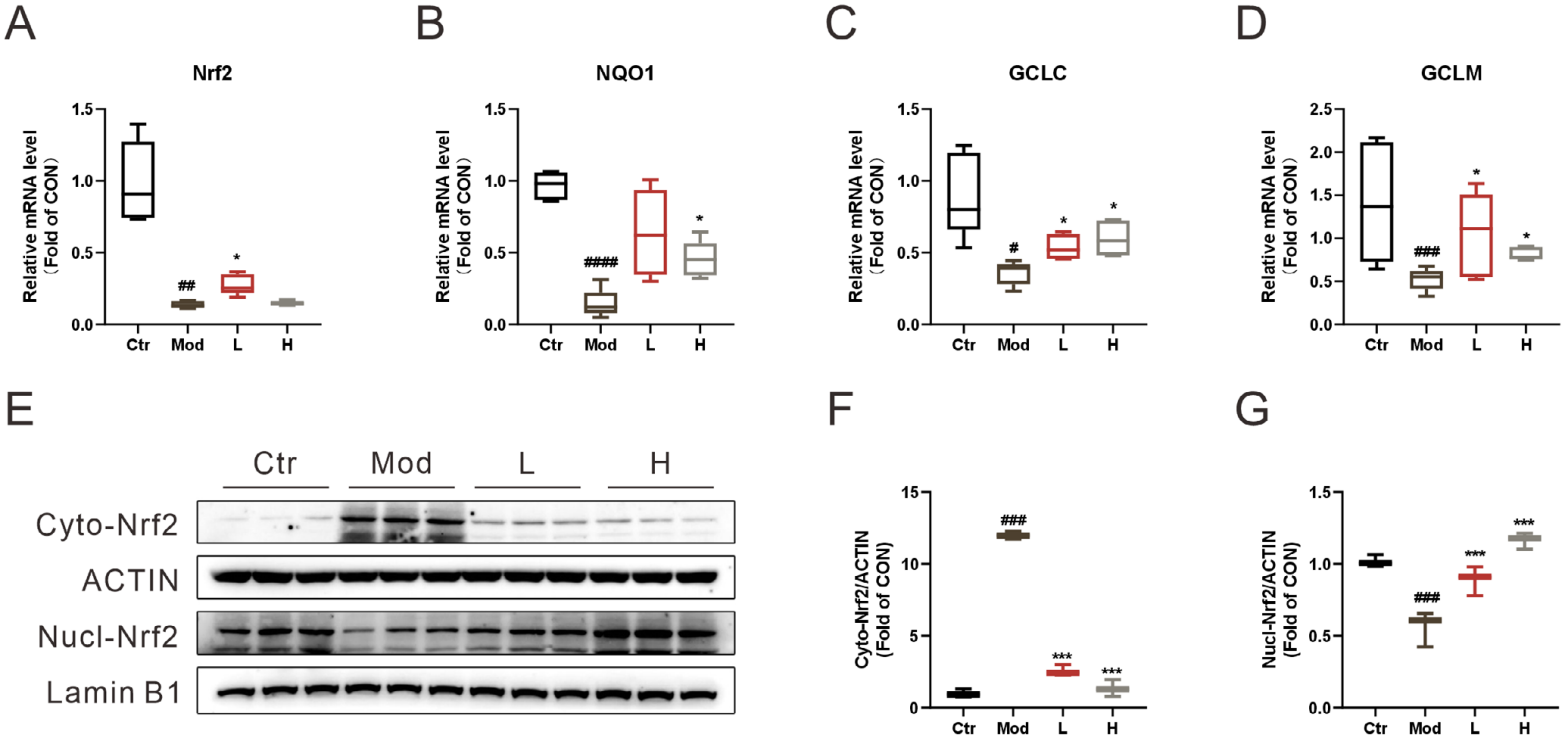
ECG prevents liver from acute liver injury in LPS‐induced mice. Serum ALT (A) and AST (B) levels; representative pathological images of liver (C); relative mRNA levels of IL‐6 (D), IL‐1β (E), and TNFα (F) in liver. Data were presented as mean ± SEM (*n* = 3–5). ^#^ *p* < 0.05, ^##^ *p* < 0.01, ^###^ *p* < 0.001 vs. the Ctr group. *p < 0.05, ***p < 0.001 vs. the Mod group.

To evaluate the effect of ECG on LPS‐induced hepatic oxidative stress, qPCR and Western blot were used to detect antioxidant‐related indicators. qPCR results showed that LPS administration reduced the mRNA expression levels of Nrf2, NQO1, GCLC, and GCLM, while ECG treatment effectively increased these levels (Figure 4A–D). WB analysis revealed that compared with the control group, cytoplasmic Nrf2 expression was significantly increased in the model group, while nuclear Nrf2 expression was significantly decreased. After ECG treatment, Nrf2 nuclear translocation was promoted, leading to increased nuclear Nrf2 expression (Figure 4E–G). These findings indicate that ECG can effectively prevent LPS‐induced hepatic oxidative stress in mice.

### 3.6 ECG Inhibits Ferroptosis in LPS‐Induced Mice

**FIGURE 5:**
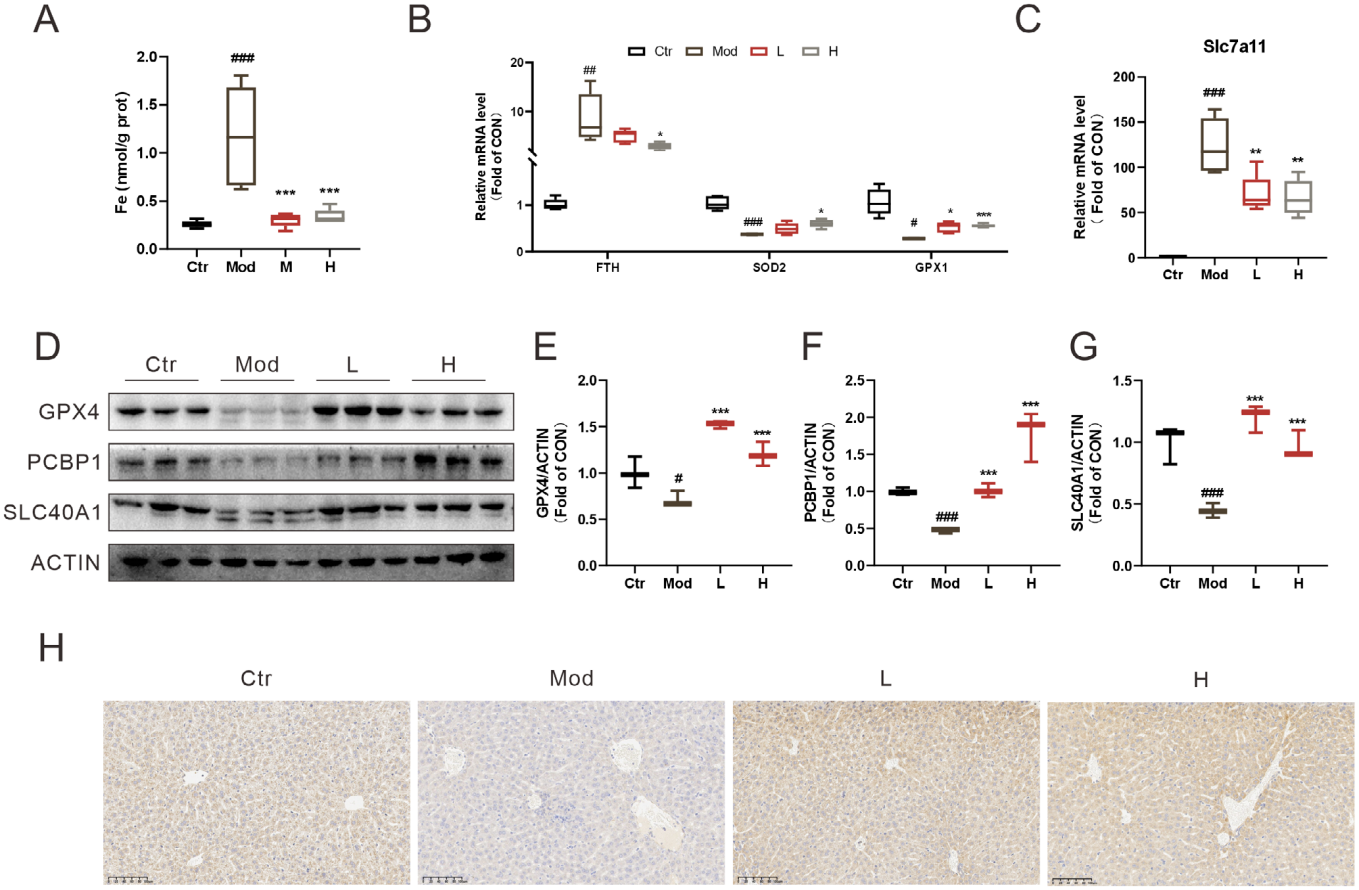
ECG inhibits ferroptosis in LPS‐induced mice. Fe content in the liver (A); relative mRNA levels of FTH, SOD2, GPX1, and *slc7a11* in the liver (B–C). Representative WB bands of GPX4, PCBP1, SLC40A1 (D); quantitative analysis of WB bands (E–G). Representative images of GPX4 immunohistochemistry in the liver (H). Data were presented as mean ± SEM (*n* = 3–5). ^#^ *p* < 0.05, ^##^ *p* < 0.01, ^###^ *p* < 0.001 vs. the Ctr group. *p < 0.05, **p < 0.01, ***p < 0.001 vs. the Mod group.

Additionally, ferroptosis is one of the pathological features of LPS‐induced ALI (Zhao et al. 2023). Consistent with expectations, we observed higher iron content in the model group, which was effectively diminished by ECG treatment (Figure 5A). Furthermore, mRNA levels of the ferroptosis markers FTH and slc7a11 were elevated in the model group compared to the control group, while the mRNA level of GPX1 and the anti‐ferroptotic enzyme SOD2 was lower than that in the control group. ECG treatment reversed these alterations (Figure 5B,C). Moreover, LPS reduced the protein levels of GPX4, PCBP1, and SLC40A1, while ECG increased those protein expressions (Figure 5D–G). Immunohistochemistry analysis results showed that LPS led to the downregulation of GPX4, whereas ECG markedly increased its expression (Figure 5H). These results indicate that ECG not only inhibits apoptosis but also counteracts ferroptosis in the liver of mice with LPS‐induced ALI.

### 3.7 Construction of an “ECG‐Ingredients–Sepsis–Targets” Network

**FIGURE 6:**
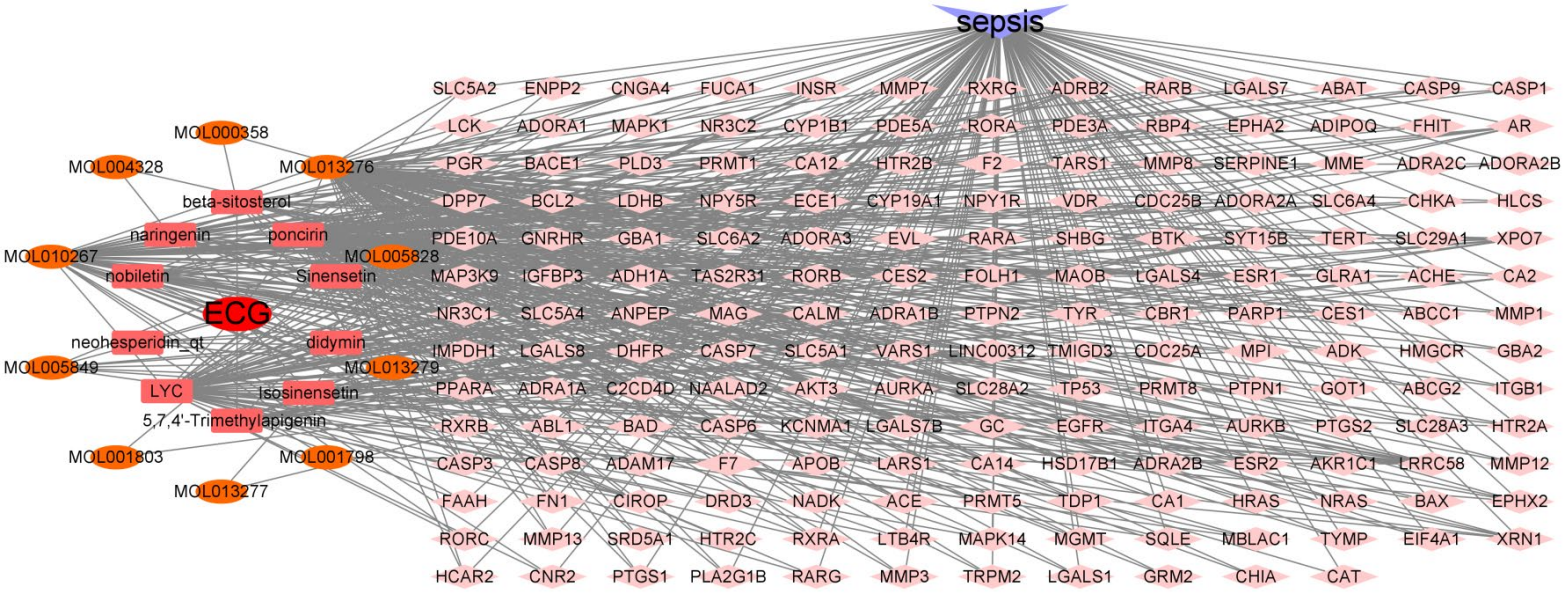
Construction of an “ECG‐ingredients‐sepsis‐targets” network.

Active ingredients of ECG were identified using the TCMSP database, leading to the isolation of 10 active components including beta‐sitosterol and naringenin (Figure 6). ECG‐related targets were compiled from TCMSP and Swiss Target Prediction, totaling 180 unique targets after removing duplicates.

### 3.8 PPI Network Construction

**FIGURE 7:**
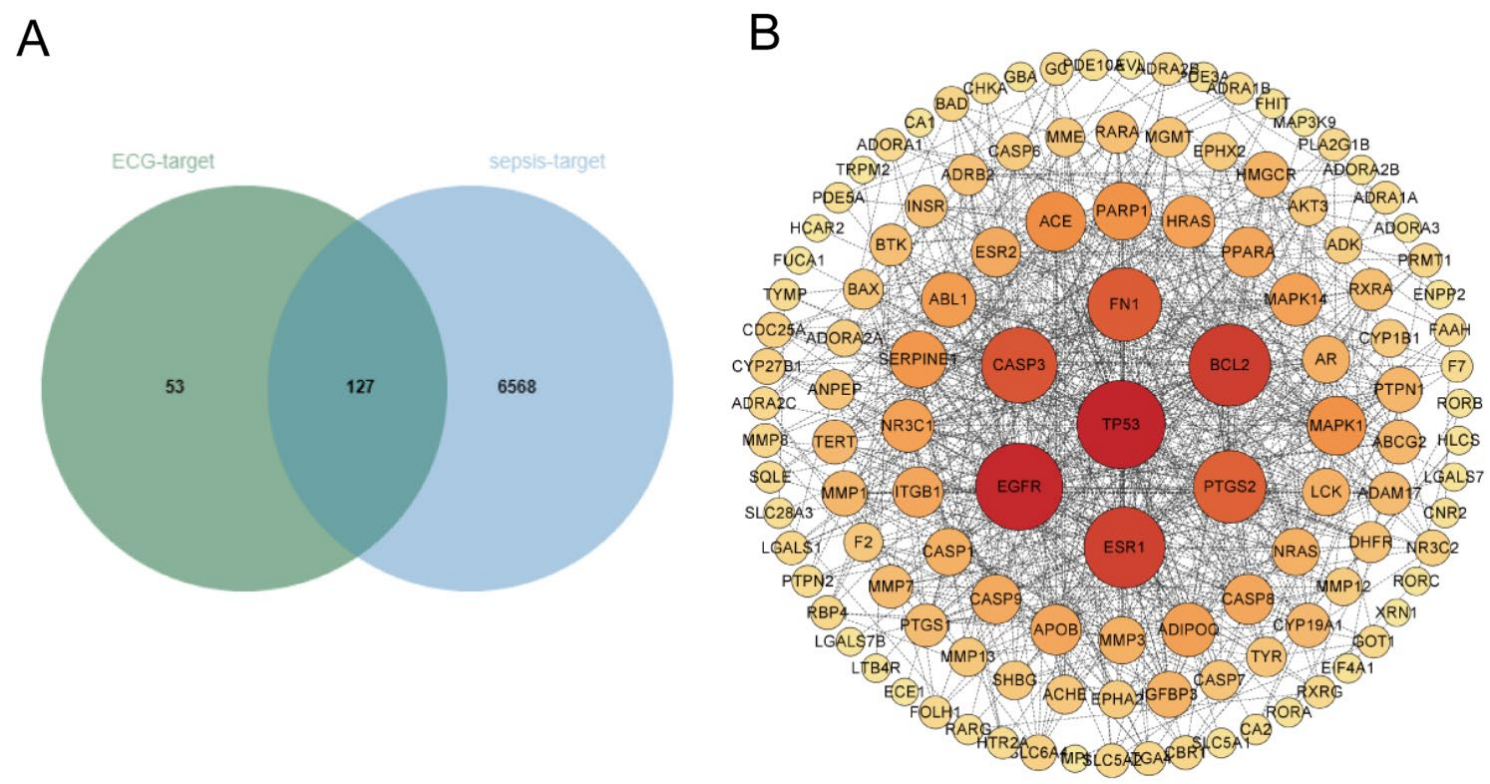
PPI network construction. Venn diagram of ECG and sepsis‐related targets (A), PPI network of potential targets (B).

A comprehensive search across multiple databases yielded 6695 sepsis‐related targets. The overlap between these sepsis‐related targets and ECG targets resulted in 127 common targets (Figure 7A). Analysis of these common targets using the STRING database highlighted key proteins such as EGFR and TP53, suggesting their significant roles in mediating the effects of ECG on LPS‐induced ALI (Figure 7B).

### 3.9 GO and KEGG Pathway Enrichment Analysis of ECG

**FIGURE 8:**
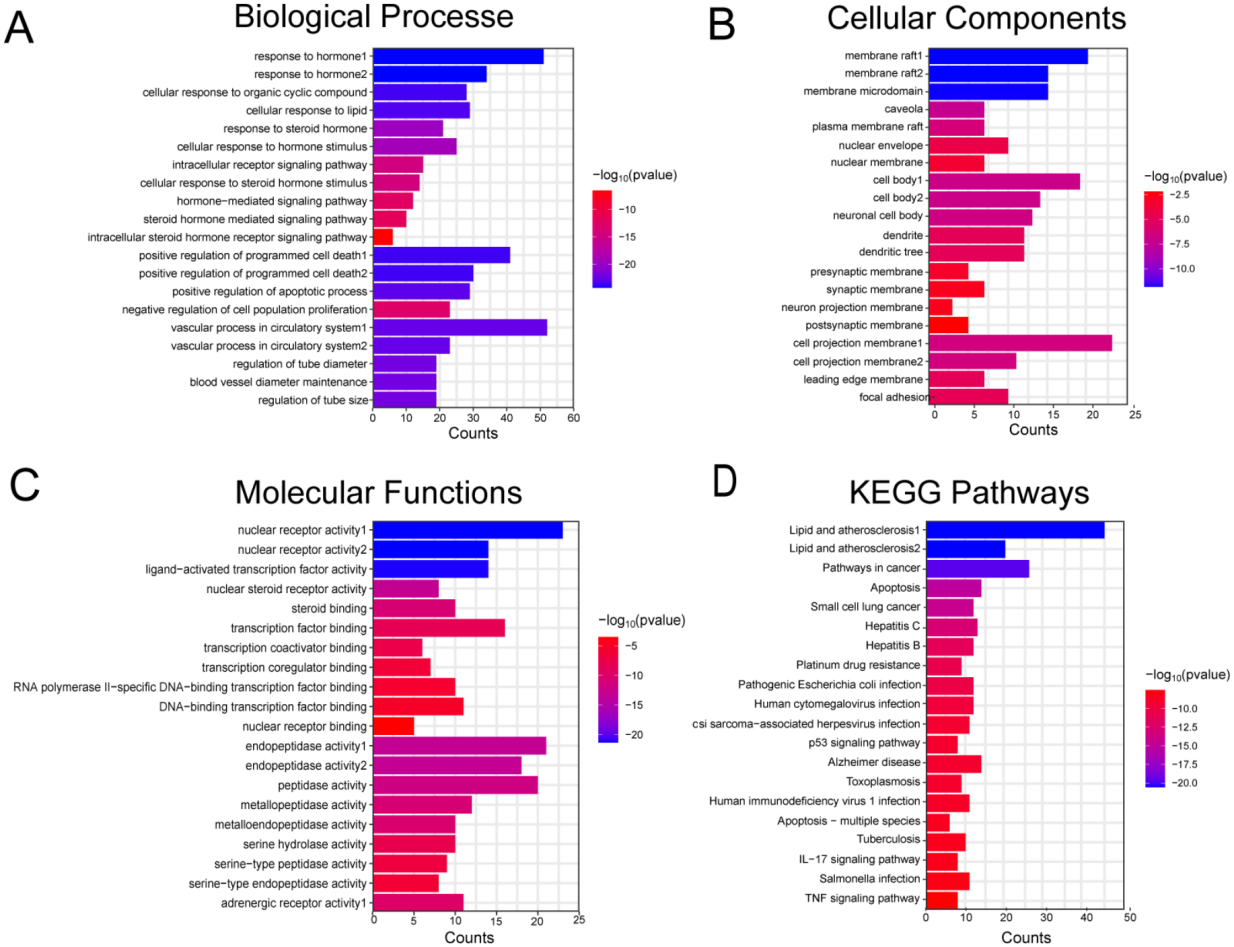
GO and KEGG pathway enrichment analysis of ECG. Biological processes (A), cellular components (B), molecular function (C), KEGG pathway (D).

GO enrichment analysis was performed on overlapping targets using the DAVID database. Biological processes implicated included responses to hormones (Figure 8A), such as hormonal signaling pathways involved in cellular responses to organic cyclic compounds and lipids. Key cellular components affected encompassed membrane structures specifically including membrane rafts, microdomains, caveolae, and plasma membrane rafts (Figure 8B), which highlight the cellular localization of ECG's targets. Molecular function analysis revealed significant involvement of nuclear receptor activity, ligand‐activated transcription factor activity, nuclear steroid receptor activity, and steroid binding (Figure 8C). These functions underscore the regulatory potential of ECG through modulation of transcription and signaling pathways. Additionally, KEGG pathway enrichment analysis further elucidated the pathways significantly associated with the mechanism of action of ECG in treating sepsis. Notably, pathways such as apoptosis, the p53 signaling pathway, the IL‐17 signaling pathway, and the TNF signaling pathway were highlighted as closely related to ECG's therapeutic effects (Figure 8D). These pathways are crucial in the regulation of inflammatory responses and cell survival, thus supporting the potential efficacy of ECG in mitigating sepsis‐related pathologies.

### 3.10 Interactions Between Active Ingredients and Sepsis Targets

**FIGURE 9:**
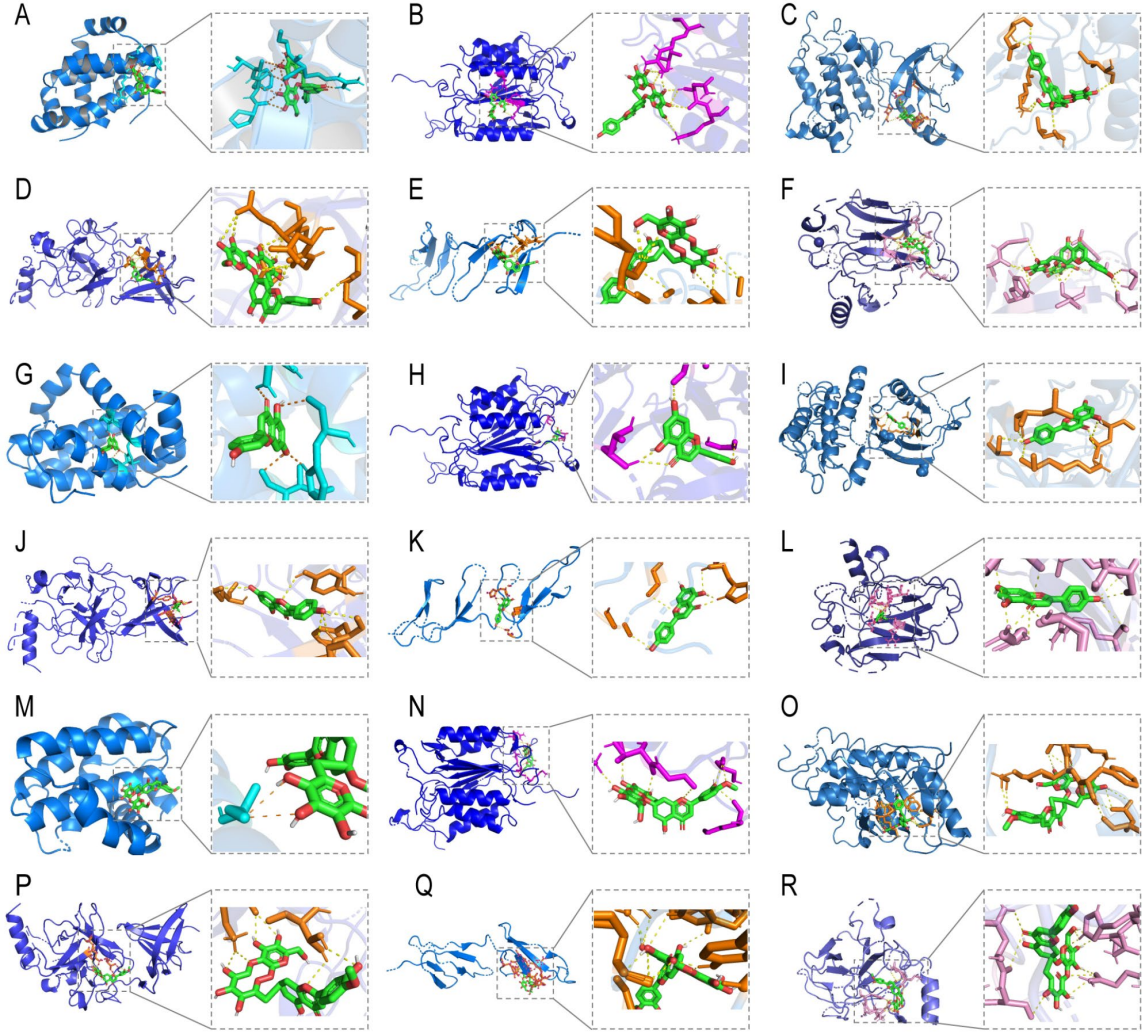
Molecular docking analysis of the bonding patterns of naringin, naringenin, and neohesperidin with key molecular targets: BCL2, CASP3, EGFR, ESR1, FN1, and TP53. The panels detail the interactions as follows: Naringin with the listed targets (A–F), naringenin with the targets (G–L), and neohesperidin with the targets (M–R).

To enhance our understanding of the pharmacological effects of active ingredients on sepsis targets, we analyzed naringin, naringenin, and neohesperidin, and associated them with 6 primary targets including BCL2, CASP3, EGFR, ESR1, FN1, and TP53 (see Table 2). The ligand‐receptor interaction diagrams are presented in Figure 9A–R. Our findings reveal the binding energies for naringin with BCL2, CASP3, EGFR, ESR1, FN1, and TP53 as −7.85, −3.41, −10.93, −10.19, −9.28, and −13.23 kcal/mol, respectively (Figure 9A–F). For naringenin, the binding energies with the same targets were −9.26, −6.64, −8.94, −7.89, −8.28, and −9.68 kcal/mol (Figure 9G–L), respectively. Additionally, neohesperidin showed binding energies of −9.08, −7.95, −10.41, −8.65, −9.11, and −10.42 kcal/mol, respectively, with these targets (Figure 9M–R). These results indicate that naringin, naringenin, and neohesperidin can directly interact with critical targets, potentially exerting significant pharmacological effects in the prevention of ALI.

**TABLE 2:** Molecular docking analysis.

Molecule name (PubChem CID)	Bind energy (PDB_ID) KJ/mol					
	BCL2 (6GL8)	CASP3 (2DKO)	EGFR (8A27)	ESR1 (3CBM)	FN1 (2CG7)	TP53 (3D06)
Naringin (442428)	−7.85	−3.41	−10.93	−10.19	−9.28	−13.23
Naringenin (439246)	−9.26	−6.64	−8.94	−7.89	−8.28	−9.68
Neohesperidin (442439)	−9.08	−7.95	−10.41	−8.65	−9.11	−10.42

## 4 Discussion

This study demonstrates that ECG exerts hepatoprotective effects against LPS‐induced ALI through multi‐target modulation of inflammatory, apoptotic, and ferroptotic pathways. While our findings align with previous reports on the efficacy of natural products in liver injury (Zhao et al. 2021; Shi et al. 2017), the mechanistic insights revealed here extend current understanding by elucidating ECG's coordinated actions across distinct cellular pathways. Below, we contextualize these results within broader biological frameworks and highlight their translational implications.

### 4.1 Anti‐Inflammatory Mechanisms: Beyond Cytokine Suppression

The observed reduction in IL‐6, IL‐1β, and TNFα levels reflects ECG's ability to disrupt early inflammatory signaling. These cytokines are pivotal in activating Kupffer cells and recruiting neutrophils, which exacerbate hepatic necrosis via ROS/RNS overproduction (Radi 2004). Notably, our network pharmacology identified TNF and IL‐17 signaling as key pathways. TNFα not only amplifies inflammation through NF‐κB but also induces hepatocyte apoptosis via caspase‐8 activation (Woolbright and Jaeschke 2017). Similarly, IL‐17 promotes neutrophil infiltration by upregulating chemokines like CXCL1 and CXCL2, as documented in prior work (Zhao et al. 2024). ECG's suppression of these cytokines likely disrupts this vicious cycle, corroborated by attenuated histopathological damage. Importantly, these findings mirror our prior work on ECG's inhibition of TLR4/MyD88/NF‐κB in LPS‐induced lung injury (Xu et al. 2024), suggesting a conserved anti‐inflammatory mechanism across organs. However, hepatic‐specific regulators such as Kupffer cell‐derived HMGB1 may further modulate ECG's effects in ALI, as reported previously (Zhang et al. 2020), warranting future investigation.

### 4.2 Apoptosis, Oxidation and Ferroptosis: Tribble Modulation of Cell Death

ECG's regulation of Bax/Bcl2 and CASP3 underscores its anti‐apoptotic role. Bax oligomerization induces mitochondrial outer membrane permeabilization (MOMP), releasing cytochrome c and activating caspase‐3 (CASP3), a hallmark of intrinsic apoptosis (Chen et al. 2021). Concurrently, ECG's normalization of GPX4 and SLC7A11 highlights its anti‐ferroptotic activity. GPX4 detoxifies lipid peroxides, while SLC7A11, which is a component of system Xc^−^ sustains glutathione synthesis, both critical for ferroptosis resistance (Wu et al. 2023). Intriguingly, p53 is a target with strong binding affinity to naringin (−13.23 kcal/mol) and orchestrates both apoptosis (via Bax transactivation) and ferroptosis (by repressing SLC7A11) (Zhao et al. 2024). This dual regulation suggests ECG may intercept shared upstream regulators like p53, offering a novel therapeutic angle distinct from single‐pathway inhibitors.

### 4.3 Network Pharmacology and Synergistic Actions

The 127 shared targets between ECG and sepsis implicate pathways such as p53 and TNF signaling, which are consistent with our experimental data. For instance, molecular docking revealed naringin's high affinity for TP53, a result that aligns with ECG's downregulation of pro‐apoptotic Bax. Similarly, neohesperidin's interaction with EGFR (−10.41 kcal/mol) may suppress EGFR/STAT3 signaling, a driver of inflammation (Chen et al. 2025). These computational predictions, validated experimentally, exemplify the “multi‐component, multi‐target” paradigm of TCM. Notably, while our earlier study highlighted ECG's NLRP3 inflammasome inhibition in lung injury (Xu et al. 2024), the current work emphasizes ferroptosis regulation in liver injury, underscoring tissue‐specific therapeutic adaptations.

### 4.4 Translational Implications and Limitations

While murine models provide valuable mechanistic insights, interspecies differences in LPS sensitivity as reported previouslly (Lozano‐Aponte et al. 2020) necessitate caution in extrapolating results to humans. Furthermore, ECG's long‐term safety and sex‐dependent effects remain unaddressed. Nevertheless, the conserved nature of pathways like p53 and TNF across species supports ECG's translational potential. Additionally, interspecies differences in drug metabolism, immune responses, and hepatic physiology necessitate further validation in human cell lines or non‐rodent models. Furthermore, ECG's traditional use in TCM for respiratory ailments (Jiang et al. 2014) supports its safety profile, but well‐designed clinical trials are essential to confirm efficacy and dosing in humans. Future studies should explore ECG's effects in combination with standard therapies to assess synergistic benefits in complex ALI etiologies.

This study adds to the growing evidence supporting the use of natural products in managing ALI, an area where effective treatments are limited. Our findings suggest that ECG, with its diverse pharmacological effects, could serve as a foundation for multi‐target drug development aimed at managing complex liver diseases. The simultaneous targeting of inflammation, apoptosis, and ferroptosis offers a unique advantage over single‐pathway drugs, which potentially provide a more robust therapeutic response with fewer side effects. Further studies, especially clinical trials, are warranted to validate ECG's efficacy in human populations and to refine dosing strategies that maximize its protective effects in ALI management. This holistic approach aligns well with contemporary integrative medicine paradigms, offering a complementary route that may enhance standard therapeutic regimens for ALI.

## 5 Conclusions

This study demonstrates that ECG effectively attenuates LPS‐induced acute liver injury (ALI) through a synergistic modulation of inflammatory, apoptotic, and ferroptotic pathways. By integrating network pharmacology with experimental validation, we elucidated ECG's multi‐target mechanisms, revealing its unique ability to simultaneously suppress cytokine storms (e.g., TNFα, IL‐6), restore apoptosis‐related proteins (Bax/Bcl2/CASP3), and inhibit ferroptosis via GPX4/SLC7A11 upregulation and iron overload normalization. Notably, the identification of p53 as a high‐affinity target for ECG's bioactive compounds (e.g., naringin) underscores its role in intercepting shared upstream regulators of hepatocyte death, offering a novel therapeutic strategy distinct from single‐pathway inhibitors.

The translational significance of this work lies in its holistic approach, which bridges traditional Chinese medicine principles with modern systems biology. ECG's multi‐component synergy aligns with the growing demand for natural products capable of addressing complex pathologies like ALI, where redundant pathways drive disease progression. Furthermore, the conserved nature of targeted pathways (e.g., TNF/NF‐κB, p53) across species enhances its potential applicability to human conditions, including drug‐induced or sepsis‐associated liver injury.

Despite these advances, several limitations warrant consideration. While murine models provide mechanistic insights, future studies should validate ECG's efficacy in human hepatocyte systems or large‐animal models to account for interspecies differences in drug metabolism and immune responses. Additionally, exploring sex‐specific effects, long‐term safety profiles, and combinatorial regimens with existing therapies (e.g., NAC for acetaminophen toxicity) could refine its clinical utility.

In conclusion, this study not only advances the understanding of ECG's hepatoprotective mechanisms but also establishes a framework for developing multi‐target natural therapeutics against ALI. The findings highlight ECG as a promising candidate for further preclinical optimization and clinical trials, potentially bridging the gap between traditional medicine and modern precision therapeutics in critical care settings.

## Author Contributions

G.Z. wrote the main manuscript text. J.L., Z.X., Z.L., and Y.C. performed data analysis. K.Z. and X.Z. prepared figures. X.W. and C.L. interpreted the data. J.H. and Z.D. conceived and designed the study. All authors reviewed and approved the final manuscript.

## Ethics Statement

This study was approved by the Ethics Committee of Guangzhou University of Chinese Medicine (No. 20240923013), Guangdong Province, China. All of the experimental procedures involving animals were conducted in accordance with the ARRIVE guidelines and approved by the Ethics Committee of Guangzhou University of Chinese Medicine.

## Consent

The authors have nothing to report.

## Conflicts of Interest

The authors declare no conflicts of interest.

## Supporting information


**Table S1:** Post hoc power analysis with Bonferroni correction for multiple comparisons.

**Figure S1:** Overview of 15 compounds of ECG identified byUHPLC‐Q‐Exactive analysis. TIC of ECG sample: Positive ion mode (A). TIC of ECG sample: Negative ion mode (B). Compounds were identified as Stachydrine (1), Cnidioside a (2), Narirutin (3), Naringin (4), Neohesperidin (5), Dihydrokaempferol (6), Poncirin (7), Bergaptol (8), Naringenin chalcone (9), Apigenin (10), Marmin (11), Bergapten (12), Isomerazin (13), Isoimperatorin (14), and Auraptene (15).

## Data Availability

All data analyzed during this study is available on www.figshare.com (doi: https://doi.org/10.6084/m9.figshare.28891511).
