# Supplementary material for: Exocarpium Citri Grandis Attenuates Lipopolysaccharide‐Induced Acute Liver Injury Through Suppression of Inflammatory, Apoptotic, Oxidative, and Ferroptotic Pathways
Source: Food Sci Nutr. 2025 Sep 26;13(10):e71012. doi: 10.1002/fsn3.71012 (PMC12474559; doi:10.1002/fsn3.71012)
Supplement: Supplementary file 1 — Table S1: Post hoc power analysis with Bonferroni correction for multiple comparisons. [file FSN3-13-e71012-s002.docx]

| **Parameter** | **Model (Mean ± SD)** | **ECG-H (Mean ± SD)** | **Cohen’s d** | **Adjusted Power (α=0.0045)** | **Conclusion** |
| --- | --- | --- | --- | --- | --- |
| ALT | 32.12 ± 5.60 | 20.45 ± 3.29 | 2.54 | 0.96 | High Power (≥0.8) |
| AST | 66.88 ± 10.42 | 32.76 ± 3.32 | 4.52 | >0.99 | High Power (≥0.8) |
| IL-6 | 36.59 ± 17.07 | 9.41 ± 2.04 | 2.28 | 0.91 | High Power (≥0.8) |
| IL-1β | 9.44 ± 3.52 | 4.14 ± 1.12 | 2.03 | 0.82 | High Power (≥0.8) |
| TNFα | 8.58 ± 2.30 | 2.49 ± 0.91 | 2.87 | 0.98 | High Power (≥0.8) |
| Fe | 1.17 ± 0.52 | 0.34 ± 0.08 | 2.23 | 0.89 | High Power (≥0.8) |
| SOD2 | 0.37 ± 0.02 | 0.61 ± 0.08 | 3.95 | >0.99 | High Power (≥0.8) |
| BAX | 1.64 ± 0.38 | 0.79 ± 0.09 | 2.31 | 0.92 | High Power (≥0.8) |
| BCL2 | 0.16 ± 0.08 | 0.85 ± 0.21 | 3.72 | >0.99 | High Power (≥0.8) |
| SLC7A11 | 8.63 ± 4.89 | 2.91 ± 0.72 | 1.88 | 0.76 | Marginal Power (Approaching 0.8) |
| FTH | 123.74 ± 30.24 | 66.45 ± 19.34 | 2.02 | 0.82 | High Power (≥0.8) |

### ****Table S1: Post-hoc Power Analysis with Bonferroni Correction for Multiple Comparisons****

#### ****Notes****

**Bonferroni Correction**: Adjusted significance level α=0.05/11≈0.0045*α*=0.05/11≈0.0045.

**Power Interpretation**:

**High Power**: ≥0.80, indicating low risk of Type II errors.

**Marginal Power**: 0.75≤Power<0.80, suggesting potential Type II error risk.

**Insufficient Power**: <0.75, requiring cautious interpretation.
