# Supplementary figures and images for: Exocarpium Citri Grandis Attenuates Lipopolysaccharide‐Induced Acute Liver Injury Through Suppression of Inflammatory, Apoptotic, Oxidative, and Ferroptotic Pathways
Source: Food Sci Nutr. 2025 Sep 26;13(10):e71012. doi: 10.1002/fsn3.71012 (PMC12474559; doi:10.1002/fsn3.71012)

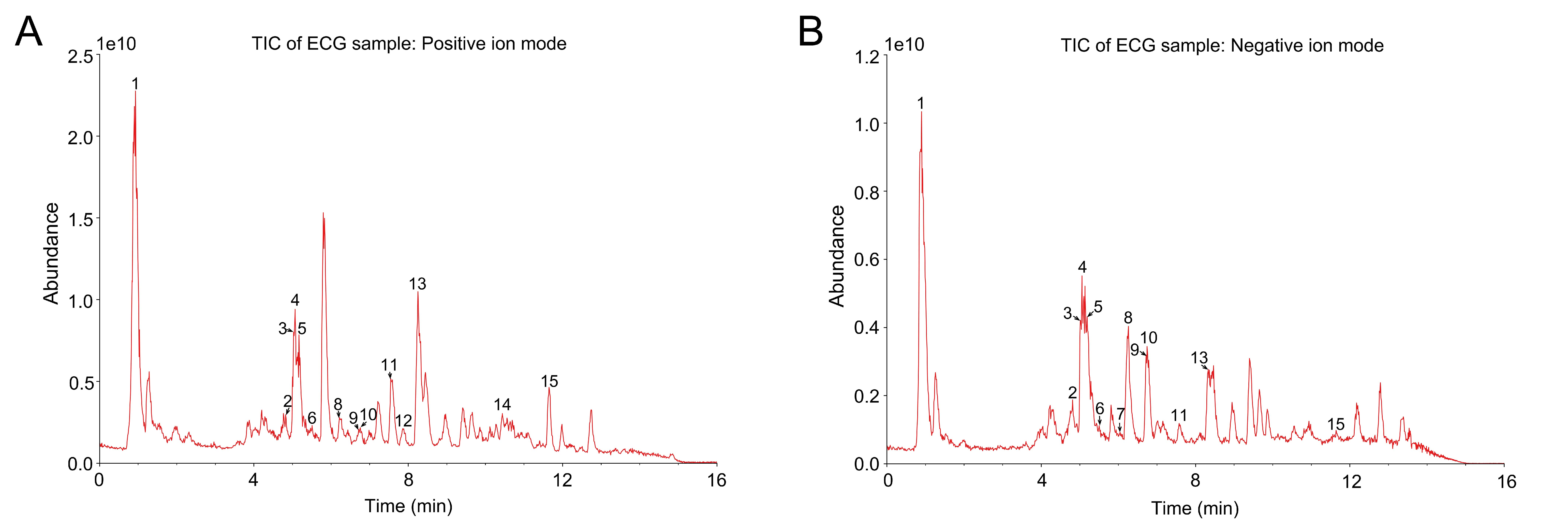

Supplement: Supplementary file 2 — Figure S1: Overview of 15 compounds of ECG identified byUHPLC‐Q‐Exactive analysis. TIC of ECG sample: Positive ion mode (A). TIC of ECG sample: Negative ion mode (B). Compounds were identified as Stachydrine (1), Cnidioside a (2), Narirutin (3), Naringin (4), Neohesperidin (5), Dihydrokaempferol (6), Poncirin (7), Bergaptol (8), Naringenin chalcone (9), Apigenin (10), Marmin (11), Bergapten (12), Isomerazin (13), Isoimperatorin (14), and Auraptene (15). [file FSN3-13-e71012-s001.tif]
